# Stereotactic iodine-125 brachytherapy for brain tumors: temporary versus permanent implantation

**DOI:** 10.1186/1748-717X-7-94

**Published:** 2012-06-19

**Authors:** Maximilian I Ruge, Philipp Kickingereder, Stefan Grau, Harald Treuer, Volker Sturm, Juergen Voges

**Affiliations:** 1 Department for Stereotaxy and Functional Neurosurgery, University of Cologne, Kerpener Straße 62, Cologne, 50937, Germany; 2 Department for Neurosurgery, University of Cologne, Kerpener Straße 62, Cologne, 50937, Germany; 3 Department of Stereotactic Neurosurgery, University of Magdeburg, Leipziger Str. 44, Magdeburg, 39120, Germany

**Keywords:** Stereotactic Brachytherapy, Permanent/Temporary Low Dose Rate Implantation, Brain Tumors

## Abstract

Stereotactic brachytherapy (SBT) has been described in several publications as an effective, minimal invasive and safe highly focal treatment option in selected patients with well circumscribed brain tumors <4 cm. However, a still ongoing discussion about indications and technique is hindering the definition of a clear legitimation of SBT in modern brain tumor treatment. These controversies encompass the question of how intense the irradiation should be delivered into the target volume (dose rate). For instance, reports about the use of high does rate (HDR) implantation schemes ( >40 cGy/h) in combination with adjuvant external beam radiation and/or chemotherapy for the treatment of malignant gliomas and metastases resulted in increased rates of radiation induced adverse tissue changes requiring surgical intervention. Vice versa, such effects have been only minimally observed in numerous studies applying low dose rate (LDR) regiments (3–8 cGy/h) for low grade gliomas, metastases and other rare indications. Besides these observations, there are, however, no data available directly comparing the long term incidences of tissue changes after HDR and LDR and there is, furthermore, no evidence regarding a difference between temporary or permanent LDR implantation schemes. Thus, recommendations for effective and safe implantation schemes have to be investigated and compared in future studies.

## 

Stereotactic implantation of irradiation sources (so called stereotactic brachytherapy (SBT)) has been applied for intrinsic brain tumors and metastases for more than four decades in numerous patients. The majority of studies reported about the application of high-dose rate (HDR) iodine-125 implants (40-70 cGy/h) for high-grade gliomas, including two prospective randomized trials, which compared standard treatment regiments with/without SBT [1,2]. This approach, however, was associated with high incidence of radiation induced adverse effects requiring repeated surgery and failed to proof any significant oncological benefit as compared to standard treatment regiments. Another approach using SBT was the application of low-dose rate (LDR) implants (3-8 cGy/h) for slow growing low-grade gliomas or brain metastases which demonstrated in several very recent publications to be associated with only little permanent deficits and almost an absence of radiation induced necrosis [3-17].

A recent comprehensive review on SBT for brain tumors by Schwarz et al. [17] summarized almost all published knowledge about this technique with the clear intention to clarify and overcome some of the preconceptions associated with brachytherapy. The authors elucidated in this well written and thoroughly investigated review the rationale, physical and biological characteristics, surgical technique, indications, complications and – most important – evidence in a critical and comprehensive manner. Especially laudable is the authors´ clear recommendation to LDR implantation schemes instead of HDR as used by US groups in treatment protocols for high grade gliomas which caused unacceptable high rates of treatment relevant radiation induced necrosis [1,18,19].

Since over two decades our group also applies stereotactic iodine-125 brachytherapy in now over 1200 patients for the treatment of - predominantly low-grade - brain tumors. Based on this experience we would like to contribute the following aspects to the discussion:

1. Schwarz et al. mention an increased risk for prolonged edema and late radiation necrosis when using permanent implantation as compared to temporary LDR implantation and refer to non human experimental data of the mid 1980´s [20-22]. Our applied prospective treatment protocol for low grade gliomas (WHO grades I and II) stipulates permanent implantation of iodine-125 seeds with initial dose rates of 0.02 – 0.03 Gy/h (prescribed surface dose: 50–65 Gy). With this strategy we very rarely observe prolonged edema or late radiation necrosis [3,4,6,7]. Interestingly, the rate of surgically relevant cysts after temporary implantation of iodine-125 seeds for low grade gliomas in a pediatric population as reported by Korinthenberg et al. [10] was three times as high (33/94 patients = 35.1 %) as in a similar population treated with permanent implants by our group (16/142 Patients = 11.3 %) [3]. Furthermore, Kreth et al. evaluated risk factors for SBT in 515 patients with low grade brain tumors and mentioned no significant difference of temporary vs. permanent LDR implantation schemes with regard to complications [23].Thus, our clinical experience as well as the reported findings in the literature does not support at all the authors´ reservation towards permanent implantation SBT.However, a critical comparison of long term results with regard to radiation induced tissue changes between permanent vs. temporary LDR implantation schemes is yet not available but necessary to ultimately clarify this controversy [24].

2. To complement the review’s physics part we may indicate that the Task Group 43 of the AAPM (American Academy of Physical Medicine) introduced (beginning in 1995) an internationally accepted standard for the dosimetry of iodine-125 seeds allowing to compare dosimetry between different countries and groups [25-30].

3. Further, we concur with Schwarz et al. on the importance of post operative imaging (either by intraoperative X-ray or by postoperative CT scanning) to confirm an accurate location of the implanted seeds, thus allowing a precise comparison with the irradiation plan at any time [31].

Ultimately, publications of this high quality keep one of oldest and most sophisticated neurosurgical technique in a vivid discussion as a minimal invasive, safe, and highly effective neuro-oncological local treatment option for selected patient populations.

## Abbreviations

SBT, Stereotactic BrachyTherapy; HDR, High Does Rate; LDR, Low Dose Rate; cGy/h, Centigray Per Hour; AAPM, American Academy of Physical Medicine.

## References

[B1] Laperriere NJ Leung PM McKenzie S Milosevic M Wong S Glen J Pintilie M Bernstein M Randomized study of brachytherapy in the initial management of patients with malignant astrocytoma Int J Radiat Oncol Biol Phys 1998 41 5 1005 1011 10.1016/S0360-3016(98)00159-X 9719109

[B2] Selker RG Shapiro WR Burger P Blackwood MS Arena VC Gilder JC Malkin MG Mealey JJ Neal JH Olson J Robertson JT Barnett GH Bloomfield S Albright R Hochberg FH Hiesiger E Green S The Brain Tumor Cooperative Group NIH Trial 87–01: a randomized comparison of surgery, external radiotherapy, and carmustine versus surgery, interstitial radiotherapy boost, external radiation therapy and carmustine Neurosurgery 2002 51 2 343 355 discussion 355–347 12182772

[B3] Ruge MI Simon T Suchorska B Lehrke R Hamisch C Koerber F Maarouf M Treuer H Berthold F Sturm V Voges J Stereotactic brachytherapy with iodine-125 seeds for the treatment of inoperable low-grade gliomas in children: long-term outcome J Clin Oncol 2011 29 31 4151 4159 10.1200/JCO.2011.37.3381 21969508

[B4] Suchorska B Ruge M Treuer H Sturm V Voges J Stereotactic brachytherapy of low-grade cerebral glioma after tumor resection Neuro Oncol 2011 13 10 1133 1142 10.1093/neuonc/nor100 21868412 PMC3177661

[B5] Kreth F Faist M Grau S Ostertag C Interstitial 125I radiosurgery of supratentorial de novo WHO Grade 2 astrocytoma and oligoastrocytoma in adults: long-term results and prognostic factors Cancer 2006 106 6 1372 1381 10.1002/cncr.21750 16470609

[B6] Voges J Treuer H Schlegel W Pastyr O Sturm V Interstitial irradiation of cerebral gliomas with stereotactically implanted iodine-125 seeds Acta Neurochir Suppl (Wien) 1993 58 108 111 8109270 10.1007/978-3-7091-9297-9_25

[B7] Voges J Sturm VV Interstitial irradiation with stereotactically implanted I-125 seeds for the treatment of cerebral glioma Crit Rev Neurosurg 1999 9 4 223 233 10.1007/s003290050137 10436211

[B8] Peraud A Goetz C Siefert A Tonn JC Kreth FW Interstitial iodine-125 radiosurgery alone or in combination with microsurgery for pediatric patients with eloquently located low-grade glioma: a pilot study Childs Nerv Syst 2007 23 1 39 46 16972111 10.1007/s00381-006-0203-7

[B9] Schnell O Scholler K Ruge M Siefert A Tonn JC Kreth FW Surgical resection plus stereotactic 125I brachytherapy in adult patients with eloquently located supratentorial WHO grade II glioma - feasibility and outcome of a combined local treatment concept J Neurol 2008 255 10 1495 1502 10.1007/s00415-008-0948-x 18677635

[B10] Korinthenberg R Neuburger D Trippel M Ostertag C Nikkhah G Long-term results of brachytherapy with temporary iodine-125 seeds in children with low-grade gliomas Int J Radiat Oncol Biol Phys 2011 79 4 1131 1138 10.1016/j.ijrobp.2009.12.040 20510544

[B11] Kreth FW Warnke PC Ostertag CB Interstitial radiosurgery of low grade glioma Nervenarzt 1993 64 10 633 639 8232676

[B12] Ostertag CB Kreth FW Iodine-125 interstitial irradiation for cerebral gliomas Acta Neurochir (Wien) 1992 119 1–4 53 61 1481753 10.1007/BF01541782

[B13] Ostertag CB Stereotactic interstitial radiotherapy for brain tumors J Neurosurg Sci 1989 33 1 83 89 2674362

[B14] Ruge MI Suchorska B Maarouf M Runge M Treuer H Voges J Sturm V Stereotactic 125Iodine Brachytherapy for the Treatment of Singular Brain Metastases: Closing a Gap? Neurosurgery 2011 68 5 1209 1219 21273930 10.1227/NEU.0b013e31820b526a

[B15] Ruge MI Kickingereder P Grau S Hoevels M Treuer H Sturm V Stereotactic biopsy combined with stereotactic (125)iodine brachytherapy for diagnosis and treatment of locally recurrent single brain metastases J Neurooncol 2011 105 1 109 118 10.1007/s11060-011-0571-z 21479963

[B16] Ruge MI Kocher M Maarouf M Hamisch C Treuer H Voges J Sturm V Comparison of stereotactic brachytherapy (125 iodine seeds) with stereotactic radiosurgery (LINAC) for the treatment of singular cerebral metastases Strahlenther Onkol 2011 187 1 7 14 10.1007/s00066-010-2168-4 21234527

[B17] Schwarz SB Thon N Nikolajek K Niyazi M Tonn JC Belka C Kreth FW Iodine-125 brachytherapy for brain tumours - a review Radiat Oncol 2012 7 1 30 10.1186/1748-717X-7-30 22394548 PMC3354996

[B18] Gutin PH Prados MD Phillips TL Wara WM Larson DA Leibel SA Sneed PK Levin VA Weaver KA Silver P External irradiation followed by an interstitial high activity iodine-125 implant "boost" in the initial treatment of malignant gliomas: NCOG study 6 G-82-2 Int J Radiat Oncol Biol Phys 1991 21 3 601 606 10.1016/0360-3016(91)90676-U 1651302

[B19] Scharfen CO Sneed PK Wara WM Larson DA Phillips TL Prados MD Weaver KA Malec M Acord P Lamborn KR High activity iodine-125 interstitial implant for gliomas Int J Radiat Oncol Biol Phys 1992 24 4 583 591 10.1016/0360-3016(92)90702-J 1429079

[B20] Groothuis DR Wright DC Ostertag CB The effect of 125I interstitial radiotherapy on blood–brain barrier function in normal canine brain J Neurosurg 1987 67 6 895 902 10.3171/jns.1987.67.6.0895 3681428

[B21] Fike JR Cann CE Phillips TL Bernstein M Gutin PH Turowski K Weaver KA Davis RL Higgins RJ DaSilva V Radiation brain damage induced by interstitial 125I sources: a canine model evaluated by quantitative computed tomography Neurosurgery 1985 16 4 530 537 10.1227/00006123-198504000-00015 3990932

[B22] Ostertag CB Brachytherapy–interstitial implant radiosurgery Acta Neurochir Suppl (Wien) 1993 58 79 84 8109309

[B23] Kreth FW Faist M Rossner R Birg W Volk B Ostertag CB The risk of interstitial radiotherapy of low-grade gliomas Radiother Oncol 1997 43 3 253 260 10.1016/S0167-8140(97)01948-8 9215784

[B24] Liu BL Cheng JX Zhang X Zhang W Controversies concerning the application of brachytherapy in central nervous system tumors J Cancer Res Clin Oncol 2010 136 2 173 185 10.1007/s00432-009-0741-y 19956971 PMC11827770

[B25] Nath R Anderson LL Luxton G Weaver KA Williamson JF Meigooni AS Dosimetry of interstitial brachytherapy sources: recommendations of the AAPM Radiation Therapy Committee Task Group No. 43. American Association of Physicists in Medicine Med Phys 1995 22 2 209 234 10.1118/1.597458 7565352

[B26] Williamson JF Coursey BM DeWerd LA Hanson WF Nath R Ibbott G Guidance to users of Nycomed Amersham and North American Scientific, Inc., I-125 interstitial sources: dosimetry and calibration changes: recommendations of the American Association of Physicists in Medicine Radiation Therapy Committee Ad Hoc Subcommittee on Low-Energy Seed Dosimetry Med Phys 1999 26 4 570 573 10.1118/1.598570 10227361

[B27] Williamson JF Coursey BM DeWerd LA Hanson WF Nath R Rivard MJ Ibbott G On the use of apparent activity (Aapp) for treatment planning of 125I and 103Pd interstitial brachytherapy sources: recommendations of the American Association of Physicists in Medicine radiation therapy committee subcommittee on low-energy brachytherapy source dosimetry Med Phys 1999 26 12 2529 2530 10.1118/1.598789 10619235

[B28] Williamson JF Butler W Dewerd LA Huq MS Ibbott GS Mitch MG Nath R Rivard MJ Todor D Recommendations of the American Association of Physicists in Medicine regarding the impact of implementing the 2004 task group 43 report on dose specification for 103Pd and 125I interstitial brachytherapy Med Phys 2005 32 5 1424 1439 10.1118/1.1884925 15984693

[B29] Kubo HD Coursey BM Hanson WF Kline RW Seltzer SM Shuping RE Williamson JF Report of the ad hoc committee of the AAPM radiation therapy committee on 125I sealed source dosimetry Int J Radiat Oncol Biol Phys 1998 40 3 697 702 10.1016/S0360-3016(97)00767-0 9486622

[B30] Rivard MJ Coursey BM DeWerd LA Hanson WF Huq MS Ibbott GS Mitch MG Nath R Williamson JF Update of AAPM Task Group No. 43 Report: A revised AAPM protocol for brachytherapy dose calculations Med Phys 2004 31 3 633 674 10.1118/1.1646040 15070264

[B31] Treuer H Klein D Maarouf M Lehrke R Voges J Sturm V Accuracy and conformity of stereotactically guided interstitial brain tumour therapy using I-125 seeds Radiother Oncol 2005 77 2 202 209 10.1016/j.radonc.2005.08.006 16209895

